# Homing Pigeons as Medical Messengers

**Published:** 1898-07

**Authors:** N. H. Haviland

**Affiliations:** Fulton, N. Y.


### HOMING PIGEONS AS MEDICAL MESSENGERS.

N. H. Haviland, M. D., Fulton, N. Y.

Until recently, in this country, little has been known of the important part the homing pigeon has taken in the affairs of men and nations. It is not my purpose in this article to give the origin of the homer; suffice it to say, that by a systematic series of crosses of several well-known varieties possessing great intelligence, endurance, and a strong homing instinct the homer of to-day was produced, in which we find combined the faculty of getting its bearings and a physical development which enables it to cover great distances.

I have a bird in my loft named Petroleum, which flew from Mississippi City to Newark, N. J., a distance of 1,092 miles in twenty-seven days, during which time he was kept a prisoner twenty-one days. This performance, however, is very rare, and from extreme distances, such as from points above 500 miles, the birds are at a great disadvantage, inasmuch as they are forced to forage for themselves, something they are not taught to do. The result is they are necessarily unreliable and slow, compared with the distance that can be made in one day. In 1885 the first homer flew 500 miles in one day; since then several have this record.

I recently purchased the fastest homer in this country. He flew from Wilmington, Del., to Newark, N. J., a distance of 100 miles, in 89 minutes, breaking all former records for speed. The Empire State Express could not do as well for an equal distance.

In establishing a loft it is best to buy of a reliable party two pairs of breeders of good stock, in order that their young may be crossed later without in-breeding. It is better to raise the youngsters in the loft from which they are to be trained, or placed there when not over six weeks old.

When a youngster is about a week old a seamless band is placed on its leg, which cannot be removed when it grows older. Each band has the number of the bird and a letter which denotes the year the bird was hatched. These bands are issued and registered by the National Federation of American Homing Pigeon Fanciers, having for its object the breeding, training, and racing of homing pigeons.

Last year this organization alone issued over 70,000 registered bands. Thus you will have an idea of the number of birds bred each year.

Much of the pleasure and usefulness of the homer depends upon the loft, which should be well ventilated and lighted, being arranged so that it may easily keep clean, and convenient of access, over the carriage barn or stable, having a floor space of about 5 by 12 feet, the height not over 6 feet. This will accommodate 20 or 30 birds. Each bird should have a perch of its own. One side of the loft should have shelves 12 inches wide and 12 inches apart; the shelves being divided into apartments 4 feet long. This is for each pair to keep house in, and rear their young, and should have a small door in a screen in front of each apartment, which could be easily removed to clean the shelves. Each pair should have two earthen nest pans 3 inches deep and 9 inches across.

One of the windows should be raised and held up by a frame about 8 inches high and as wide as the window.

From the top of the frame stiff wire rods should be hung two inches apart, being arranged in such a manner that they will only swing inward, thus allowing the birds from the outside to enter easily, while the egress of those that are within is effectually prevented, except when wishing to let them out, by pulling a cord and lifting all the wires out of the way. The wires are called bolting or bob wires, and can be arranged to ring an electric bell in the house or office when a bird enters. Just inside I have a trap or cage in which the bird is a prisoner till the message is removed, after which a door is opened and he is then allowed to enter the loft. The birds, except the breeders, should be given their liberty in the morning before feeding. After they have flown around for half an hour or so they will eagerly push through the bob wires to get their breakfast. Having eaten, they are kept prisoners until supper time, when they are turned out as before, and on their return are fed with grain the same as common fowls; this insures prompt return after exercise. Birds thus trained will go immediately into the loft when returning with a message.

When the birds are about two months old, and have flown around their loft, and are well acquainted with the location, they should be taken in a large picnic basket a few blocks from home and liberated—and every few days the distance should be doubled from the last liberating point.

My first use of the homing pigeon as a medical messenger was a number of years ago while attending a child, who was very sick with cholera infantum, nine miles away from home, without telegraph or telephone communication. Leaving my birds at each visit the mother gave me full reports of the case during the intervals of my calls.

One day my feathered messenger brought me word that the child was better, and that it would not be necessary for me to call till next day.

Just at dark another bird arrived with a note stating the child was worse, and to come immediately.

The mother believes the bird was the means of saving the life of her child. She also stated that it was a relief to her to have the little messengers with her, and she was lonely when the last bird had flown. Another time when my birds served me well was when my brother was sick with pneumonia, living a distance of thirty-five or forty miles, although it was during the cold days of December. They kept me regularly informed of the condition of my patient; without them it would have been impossible to have treated the case so far away.

In attending obstetrical cases in the country my man sees that I have my basket of homers the same as he does my obstetrical case. If I find my patient is suffering with false pains and my service is not required, I dispatch a bird home, stating that I will be there soon and for office patients to wait.

The remainder of the birds are left with the patient in order that she may inform me as to her condition.

When my patients keep homers, at my first visit I leave a basket of my birds and take a basket of theirs. By means of their liberating my birds, and I liberating theirs, it is possible to carry on a correspondence. Thus letters may be received and medicine sent for several days.

In like manner Dr. Lang, of Meridian, who also uses homers in his practice, exchanged a basket of birds with me, he releasing one of my birds with a letter, I replying with one of his. In this way we transacted some important business, though the distance was seventeen or eighteen miles.

The birds are useful in acute diseases, fevers, etc., when wishing to hear from patients between visits. Then, too, they are a means of pleasure, for your families can use them while away from home, visiting or camping.

The message can be written on a powder paper and folded into a narrow flat strip, about an inch and a half long, and inserted underneath the register band and the top of the paper bent or folded over the band.

To the homœopathic physician situated as I am, in a village surrounded by small towns, from two to eight miles out, and each containing a few homœopathic families, and only an allopathic doctor, for one to visit these places in each direction, and attend to his village work at times is impossible; but by aid of the homer he is enabled to do the work very much better. It is my belief that the physician should have some fad or hobby giving him rest or recreation, at the same time it is well calculated to give rest and recuperate the nerve forces. It does not take him away from home, nor does it cause a great outlay of money.

In fact, we enjoy a small vacation each day while visiting our pets. It has always been my impression that the American idea of vacation is wrong. Working with an electric push as we do for eleven and a half months, then instead of seeking a much-needed rest, we start out to see everything and everybody, and the consequence is, we are apt to return home completely tired out. My idea is to gain comfort each day, catch the sunshine, and behold the beautiful flowers by the wayside, for we are passing this way but once.

